# Deviations from normal bedtimes are associated with short-term increases in resting heart rate

**DOI:** 10.1038/s41746-020-0250-6

**Published:** 2020-03-23

**Authors:** Louis Faust, Keith Feldman, Stephen M. Mattingly, David Hachen, Nitesh V. Chawla

**Affiliations:** 1 Department of Computer Science & Engineering, University of Notre Dame, Notre Dame, IN USA; 2 Center for Network and Data Science (CNDS), University of Notre Dame, Notre Dame, IN USA; 3 Department of Sociology, University of Notre Dame, Notre Dame, IN USA

**Keywords:** Quality of life, Risk factors

## Abstract

Despite proper sleep hygiene being critical to our health, guidelines for improving sleep habits often focus on only a single component, namely, sleep duration. Recent works, however, have brought to light the importance of another aspect of sleep: bedtime regularity, given its ties to cognitive and metabolic health outcomes. To further our understanding of this often-neglected component of sleep, the objective of this work was to investigate the association between bedtime regularity and resting heart rate (RHR): an important biomarker for cardiovascular health. Utilizing Fitbit Charge HRs to measure bedtimes, sleep and RHR, 255,736 nights of data were collected from a cohort of 557 college students. We observed that going to bed even 30 minutes later than one’s normal bedtime was associated with a significantly higher RHR throughout sleep (Coeff +0.18; 95% CI: +0.11, +0.26 bpm), persisting into the following day and converging with one’s normal RHR in the early evening. Bedtimes of at least 1 hour earlier were also associated with significantly higher RHRs throughout sleep; however, they converged with one’s normal rate by the end of the sleep session, not extending into the following day. These observations stress the importance of maintaining proper sleep habits, beyond sleep duration, as high variability in bedtimes may be detrimental to one’s cardiovascular health.

## Introduction

Prolonged inadequate sleep habits have been repeatably linked to serious medical conditions such as heart disease, obesity and decreased life expectancy^1–3^. As such, proper sleep hygiene has become a key component in managing personal health and wellness. To improve our sleep habits, we are often recommended to achieve 7–9 h of sleep per night^4^; however, this goal alone neglects a less understood, yet critical aspect of proper sleep: bedtime regularity. Prior research has observed that large differences in bedtime regularity, even after adjusting for bedtime duration, are linked to worsened health outcomes, particularly for our cognitive and metabolic systems^5–7^. The literature has yet to address, however, what these disparities in bedtime regularity mean for our cardiovascular health. As such, this manuscript serves to address this gap: specifically, examining how adherence to a normal bedtime is associated with resting heart rate (RHR).

A body of literature has shown RHR to be an important biomarker for cardiovascular health^8–13^. A meta-analysis conducted by Zhang et al.^9^, including 46 studies involving 1,246,203 patients, observed that higher RHR was independently associated with increased risk of all-cause and cardiovascular mortality after adjusting for traditional risk factors^9^. In addition to mortality, a study by Cooney et al.^13^, following over 20,000 healthy men and women, found elevated RHR was a significant independent risk factor for likelihood of developing cardiovascular disease.

In addition to high RHR being a significant predictor of cardiovascular disease and mortality, studies have further shown that it is the changes in RHR over time that are associated with these outcomes^10,11^. In a meta-analysis published in the European Heart Journal, several studies focused on reducing RHR over time through the use of beta-blockers and calcium channel blockers, finding the reductions in RHR were associated with reductions in cardiovascular mortality^10^. A prominent work recently published in Open Heart observed that individuals with significant increases in RHR over time were at higher risk for all-cause and cardiovascular mortality^11^, finding every beat per minute increase was associated with a 3% higher risk for all-cause mortality, 1% higher risk for cardiovascular disease and 1% higher risk for coronary heart disease. The authors also highlighted the clinical utility in capturing trends in RHR, rather than relying on a single measure.

Given the importance of RHR as a biomarker for cardiovascular health, a better understanding of the risk factors associated with RHR is necessary for the proper interpretation of changes in RHR over time. As such, we examined bedtime deviations as a potential risk factor for elevated RHR. This was accomplished through two research questions: **RQ1**—Are deviations in bedtimes, relative to one’s normal bedtime, associated with increases in resting heart rate? and **RQ2**—How long does one’s resting heart rate take to return to baseline, following bedtime deviations?

## Results

### Participant characteristics

Table 1 provides an overview of our study cohort broken into three categories: demographics, behaviors and sleep characteristics. As the participants involved in this study were followed for multiple semesters, mutable attributes are presented for each semester, corresponding to repeated surveys. A summary of these demographics and their relationship with RHR is available in Supplementary Table 5.

**Table 1:** Overview of study cohort demographics, behaviors and sleep characteristics across duration of study.

	Wave 1 (Fall 2015)	Wave 2 (Spring 2016)	Wave 3 (Fall 2016)	Wave 4 (Spring 2017)	Wave 5 (Fall 2017)	Wave 6 (Spring 2018)	Wave 7 (Fall 2018)
Demographics							
Age, *µ* *±* *σ*	17.90 ± 0.47 years (when first entering the study)						
Sex, *n* (%)							
Male	277 (50)						
Female	280 (50)						
Ethnicity, *n* (%)							
White	360 (65)						
Latino	74 (13)						
Black	35 (6)						
Asian	51 (9)						
Other	1 (<1)						
Foreign	36 (6)						
Behaviors							
Caffeine consumption, *n* (%)							
Not at all	89 (20)	112 (22)	77 (20)	84 (24)	72 (21)	68 (23)	48 (19)
Less than 1–2 times a month	60 (14)	63 (12)	30 (8)	24 (7)	35 (10)	21 (7)	16 (6)
1–2 times a month	71 (16)	44 (9)	46 (12)	35 (10)	41 (12)	26 (9)	36 (14)
1–2 times a week	104 (24)	119 (23)	73 (19)	61 (17)	51 (15)	50 (17)	37 (15)
3 times a week or more	118 (27)	172 (34)	155 (41)	147 (42)	145 (42)	137 (45)	117 (46)
Alcohol consumption, *n* (%)							
Not at all	177 (40)	150 (30)	94 (25)	100 (28)	85 (25)	52 (17)	37 (15)
Less than 1–2 times a month	109 (25)	58 (11)	51 (13)	36 (10)	49 (14)	27 (9)	22 (9)
1–2 times a month	93 (21)	78 (15)	57 (15)	44 (13)	56 (16)	56 (19)	59 (23)
1–2 times a week	56 (13)	197 (39)	144 (38)	149 (42)	131 (38)	128 (42)	101 (40)
3 times a week or more	7 (2)	25 (5)	34 (9)	22 (6)	23 (7)	39 (13)	35 (14)
Physical activity							
[0, 1) h	115 (26)	79 (19)	108 (25)	73 (20)	51 (16)	32 (10)	19 (10)
[1, 2) h	160 (36)	161 (38)	159 (37)	146 (40)	141 (44)	135 (44)	77 (39)
[2, 3) h	90 (20)	94 (22)	81 (19)	83 (22)	88 (27)	93 (30)	71 (36)
>3 h	76 (17)	88 (20)	79 (18)	66 (18)	44 (14)	45 (15)	28 (14)
Sleep characteristics							
Duration, *n* (%)							
<6 h	15 (3)	16 (4)	13 (3)	5 (1)	5 (2)	3 (1)	3 (2)
[6, 7) h	165 (37)	143 (34)	105 (25)	78 (21)	60 (19)	57 (19)	35 (18)
[7, 8) h	216 (49)	209 (50)	239 (56)	206 (56)	200 (62)	177 (58)	110 (56)
[8, 9) h	43 (10)	52 (12)	64 (15)	71 (19)	57 (18)	62 (20)	44 (23)
>9 h	2 (0)	2 (0)	3 (1)	8 (2)	2 (1)	4 (1)	3 (2)
Normal bedtime, *n* (%)							
Before 11 pm	5 (1)	4 (1)	3 (1)	5 (1)	3 (1)	5 (2)	4 (2)
[11 pm, 12 am)	24 (5)	26 (6)	24 (6)	24 (7)	24 (7)	27 (9)	16 (8)
[12 am, 1 am)	126 (29)	119 (28)	137 (32)	113 (31)	94 (29)	88 (29)	66 (34)
[1 am, 2 am)	187 (42)	162 (38)	157 (37)	135 (37)	127 (39)	105 (34)	65 (33)
[2 am, 3 am)	75 (17)	83 (20)	80 (19)	57 (15)	51 (16)	50 (16)	28 (14)
After 3 am	24 (5)	28 (7)	23 (5)	34 (9)	25 (8)	30 (10)	16 (8)

In Table 2, we also provide the number of instances of each bedtime deviation and overall percentages. We also note that on time is defined as occurring within ±30 min of an individual’s normal bedtime. Therefore [1, 30) min could also be thought of as [31, 60] min outside the individual’s median bedtime.

**Table 2:** Number of instances for each bedtime deviation category (%).

≥3 h earlier	[2, 3) h earlier	[1, 2) h earlier	[30, 60) min earlier	[1, 30) min earlier	On time	[1, 30) min later	[30, 60) min later	[1, 2) h later	[2, 3) h later	≥3 h later
2211 (1)	5015 (2)	18,403 (7)	20,626 (8)	32,753 (13)	83,766 (33)	33,308 (13)	23,251 (9)	24,796 (10)	9087 (4)	2520 (1)

### Bedtime deviations and resting heart rate (RQ1)

For earlier bedtimes, we observe, on average, higher RHRs the earlier one goes to bed relative to their normal bedtime. Referring to Table 3, no significant differences were observed in RHR for bedtimes within 30 min of one’s normal bedtime. However, for all bedtimes earlier than 30 min, we observe significant increases in RHR. For the following day, we observe no significant differences in RHR regardless of earliness of bedtime compared to going to bed on time, with the exception of the [1, 30) min category (Table 4). Regarding later bedtimes, we again observe, on average, higher RHRs the later one goes to bed relative to their normal bedtime (Table 3). However, bedtimes within even 1–30 min later show significant increases in RHR and are exacerbated as the deviation increases. Further, we observe that increased RHR persists into the following day, remaining significant across each of the bedtime deviation categories (Table 4).

**Table 3:** Within-person differences in average resting heart rate during sleep across bedtime deviations.

Bedtime deviation	Earlier bedtimes^a,b^	Later bedtimes^b,c^
	Coeff (95% CI)	Coeff (95% CI)
On time	Reference	Reference
[1, 30) min	0.03 (−0.04, 0.10)	0.18 (0.11, 0.26)
[30, 60) min	0.24 (0.15, 0.33)	0.57 (0.47, 0.66)
[1, 2) h	0.59 (0.49, 0.69)	1.11 (1.01, 1.22)
[2, 3) h	1.10 (0.93, 1.27)	2.01 (1.84, 2.16)
*≥*3 h	2.67 (2.42, 2.93)	2.74 (2.48, 3.01)

^a^*N* = 557 participants, 153,385 nights.

^b^Coefficients are representative of differences in resting heart rate bpm adjusting for sleep duration, naps, sex, prior day’s physical activity and frequency of caffeine and alcohol consumption.

^c^*N* = 557 participants, 166,292 nights.

**Table 4:** Within-person differences in average resting heart rate during the next day across bedtime deviations.

Bedtime deviations	Earlier bedtimes^a,b^	Later bedtimes^b,c^
	Coeff (95% CI)	Coeff (95% CI)
On time	Reference	Reference
[1, 30) min	−0.09 (−0.19, 0.002)	0.07 (−0.02, 0.17)
[30, 60) min	−0.04 (−0.16, 0.07)	0.21 (0.09, 0.32)
[1, 2) h	0.08 (−0.05, 0.21)	0.35 (0.24, 0.47)
[2, 3) h	0.16 (−0.07, 0.40)	0.62 (0.44, 0.79)
*≥* 3 h	0.31 (−0.05, 0.68)	0.91 (0.58, 1.23)

^a^*N* = 557 participants, 131,079 days.

^b^Coefficients are representative of differences in resting heart rate bpm adjusting for sleep duration, naps, sex, prior day’s physical activity and frequency of caffeine and alcohol consumption.

^c^*N* = 557 participants, 141,511 days.

### Return to baseline (RQ2)

**Fig. 1:**
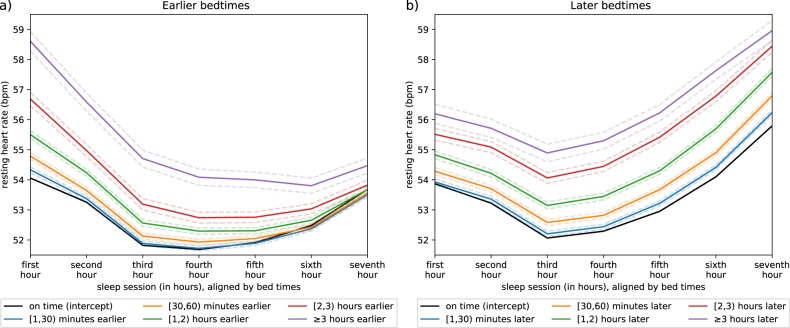
Resting heart rate across bedtimes. The panels show the hourly differences in resting heart rate across an average sleep session stratified by bedtime. **a** Resting heart rate curves as one goes to bed earlier than normal; **b** curves as one goes to bed later than normal.

**Fig. 2:**
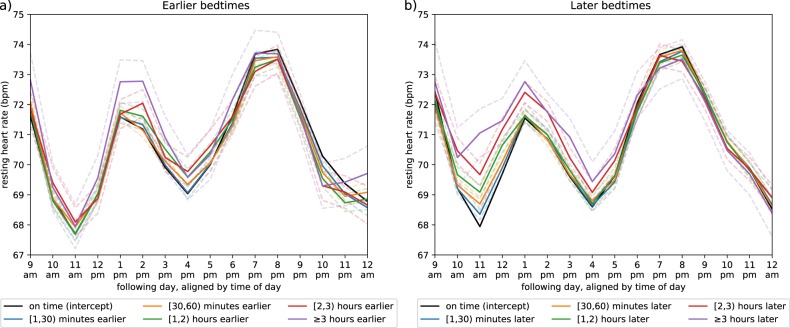
Resting heart rate across the following day. The panels show the hourly differences in average resting heart rate across the following day stratified by bedtime. **a** Resting heart rate curves as one goes to bed earlier than normal; **b** curves as one goes to bed later than normal.

Moving to our second research question (RQ2), we evaluated time for RHR to return to baseline following a bedtime deviation using a series of linear mixed effects models. A single mixed effects model was fit for each hour of sleep and consecutive hour awake up until midnight of that day. This approach allowed us to examine each hour independently and capture nonlinear trends in RHR over time. Again, we note a comprehensive description is provided in the Methods section. Considering these associations at the hourly level, for earlier bedtimes, differences in RHR are most distinct across the first few hours and slowly re-converge across one’s sleep session (Fig. 1a). We also note that by the seventh hour of sleep, on time bedtimes appear to have a higher RHR than earlier bedtimes. Regarding the following day, no significant patterns were observed (Fig. 2a). For details on hourly level coefficients, we refer the reader to Supplementary Tables 6 and 7. Regarding hourly level differences for later bedtimes, the increased RHR persists across one’s entire sleep session, remaining significantly different at the seventh hour mark, suggesting differences in RHR do not typically converge over one’s sleep session (Fig. 1b). Moving into the following day, we find these differences persist until roughly 6:00 pm at which time RHRs begin to re-converge (Fig. 2b). Again, we note hourly level coefficients can be found in Supplementary Tables 6 and 7.

Finally, as it is reasonable to assume bedtime deviations may occur in succession, such as Friday and Saturday night, we performed an additional analysis to assess whether these observations would manifest after a single bedtime deviation. We observed that bedtime deviations, even after a night with a regular bedtime, were still associated with increases in RHR across the sleep session and into the following day. These observations suggest that increases in RHR are associated with an isolated deviation from one’s normal bedtime. Details for this analysis can be found in the supplementary materials: Supplementary Methods 1.

## Discussion

The observations from this work suggest deviations from an individual’s normal bedtime may prohibit RHR from slowing to its normal pace, resulting in a higher RHR throughout one’s sleep session. Further, this short-term change to RHR may persist into the following day, with RHR returning to its normal pace by early evening. However, this extension only manifests when individuals go to bed later than their normal bedtime as opposed to earlier. While previous studies have shown pharmacological interventions and lifestyle factors such as BMI and endurance training can affect RHR, to our knowledge, this is the first study to show that variability in bedtime (after controlling for sleep duration and circadian effects) may also affect RHR^10,13–15^. It is important to note that inferring causality was outside the scope of this work. Therefore, we are unable to address the physiological association between bedtime deviations and changes in RHR. Particularly the higher RHR observed in the last hour of sleep by those going to bed on time compared to those going to bed slightly earlier. However, it remains an important question for future studies.

Further, despite RHR functioning as a useful predictor of cardiovascular health, the best practices for implementing the knowledge of RHR in clinical settings remain an open question^12^. Physicians often recommend the adoption of healthy behaviors such as adequate sleep and physical activity which in turn may lower RHR. However, the notion of determining and recommending “optimal” target RHRs requires further investigation^12^.

Regarding bedtime as a primary component of sleep hygiene, our observations align with work that shows poorer sleep hygiene, specifically variability around bed and wake times, can be detrimental to one’s health (for review, see ref. ^16^). While previous work on healthy participants relies heavily on subjective measures, variability around bedtime has been shown to be associated with decreased sleep quality^17^, increased daytime sleepiness^18^, poorer lifestyle (e.g. increased alcohol consumption, fewer nights of sufficient sleep, etc.)^19^, and poorer sleep quality^20^. Interestingly, our results align with other findings that show timing of sleep and regularity of sleep (rather than duration) is important for academic performance^21,22^. However, improving sleep hygiene may or may not influence physiology in the short term^23^.

The observations in this work further mirror investigations into the relationship between heart rate variability (HRV) and sleep. While HRV was not captured among this population, HR and HRV are physiologically and mathematically related: with higher HRV generally observed among lower HR^24^. This is an important relationship to note, as higher HRV in the high frequency spectral band is associated with improved subjective and objective measures of sleep as well as physical and mental health^15,25^. In addition, high RHR and low HRV independently contribute to mortality^10,12,26^.

With ties to physical and mental health, our observations further stress the importance of maintaining proper sleep hygiene. Given the association to RHR, over time, high variability in bedtimes, such as those among shift workers, frequent travelers, or even those staying up later on weekends, could result in changes to baseline RHR as high bedtime variability becomes a trait of one’s chronic sleep behavior. On the other hand, reducing variability and maintaining consistent bedtimes may lead to modest reductions in RHR, suggesting sleep hygiene as an additional factor to consider regarding cardiovascular health.

All participants in this study were among the same age and observed in the same environment. Therefore, additional studies across different age groups and backgrounds are necessary before determining the extent to which these results can be generalized. In particular, this research would benefit from being conducted on a non-student population, as well as specifically investigating the association between bedtime deviations and HRV, as this is another important biomarker for health and mortality.

Further, it is important to note the limitations of the devices used in the study. While Fitbits have previously been shown to provide reasonable estimates of sleep onset and offset in free living conditions, recent comparisons to gold-standard polysomnography tests have shown Fitbits to overestimate total sleep time by more than 10%^27–30^. Extending this to bedtimes, while no studies to date have specifically investigated the accuracy of bedtimes, it can be reasonably assumed that this overestimation is primarily the result of estimating bedtimes as earlier than normal and wake times as later than normal. Despite these errors in estimation, evidence from studies suggest Fitbit devices may provide similar measures for time in bed and time sleeping compared to research-grade accelerometers^30^. Further, as our study focuses on deviations within an individual, we are primarily focused on the internal consistency within an individual’s day-to-day measurements, which has been shown to be reliable^31^.

Moreover, we note our comparisons of RHR among demographics and behaviors (see Supplementary Table 5) still fall in line with prior observations. Specifically, females have, on average, higher RHR^32^ and RHR increases with the consumption of caffeine and alcohol^32,33^. Additionally, the sinusoidal curves in RHR resulting from circadian rhythm (Fig. 2) follow the same pattern as the clinical-grade wearable devices utilized in ref. ^34^.

## Methods

### Study objectives

To address the research questions outlined in the introduction of this manuscript, we utilized a data set comprised of 255,736 nights of sleep across 557 participants. Sleep and HR data were measured utilizing Fitbit Charge HRs, providing highly granular measurements of RHR and reasonable estimates of sleep duration^27^. The minimally invasive nature of these sensors allowed us to capture uninterrupted streams of participants RHR: providing measures across an individual’s entire night’s sleep and throughout the following day. This continuity allowed for a comprehensive study around not only how RHR may change following bedtime deviations, but also the average time window needed for RHR to return to baseline.

### NetHealth study

The data utilized in this manuscript come from the NetHealth study conducted at the University of Notre Dame. The study followed college students for up to 4 years, beginning the data collection in the summer of 2015 and ending in May of 2019. The most recent snapshot of the data, however, included data only up to the Fall 2019 semester. Data collection included demographic, psychometric, social network and physical activity data for the purpose of modeling the coevolution of health behaviors and social networks^35^. Participants’ sleep, heart rate and physical activity were monitored using Fitbit Charge HRs. Upon entering the study, participants were provided a Fitbit, along with a username and password to create their account with. This allowed the investigators to pull data directly from each account through Fitbit’s web API and store the data on a university server. To collect demographic and behavioral data, surveys were administered once per semester that students could complete online. To ensure completeness in the data collected, participants were asked to wear their Fitbit as much as possible and sync their device every 4−7 days.

### Ethics

This observational study was approved by the University of Notre Dame’s IRB after a full board review under protocol number 17-05-3912. All participants provided written informed consent prior to taking part in the study.

### Participants

Participants included 698 individuals who entered the university as first-year students over the course of the 2015–2016 academic year. The NetHealth cohort was split across three recruitment phases or “tiers” outlined below.

- Tier 1: A total of 391 tier 1 participants were recruited via interest surveys, e-mail, and a Facebook page in June 2015. Selection was on a first come, first served basis in keeping with the overall demographic distributions of the university.
- Tier 2: Next, 97 tier 2 participants were then recruited in November and December 2015, nominated by existing participants in the study.
- Tier 3: Finally, 210 tier 3 participants were recruited via e-mail in April 2016.

**Fig. 3:**
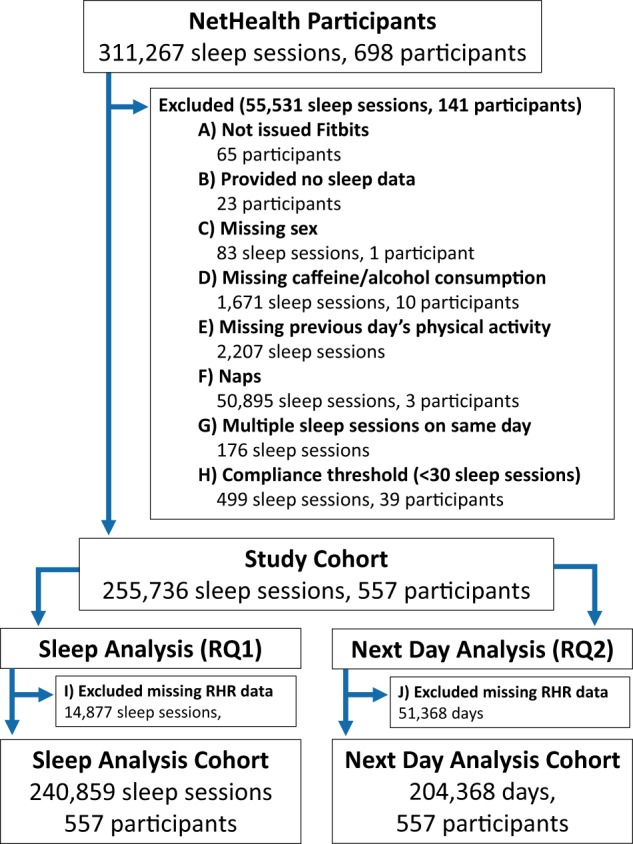
Flow diagram. Outline of cohort selection and data pre-processing steps with the number of participants/data points removed at each step.

Despite enrolling a total of 698 participants, not all were eligible for analysis. A total of 65 participants were excluded as they were not issued Fitbits (Fig. 3A), with reasons ranging from participants declining them to dropping the study before the device could be issued. Among the participants who received Fitbits, an additional 23 were excluded (Fig. 3B), as they did not wear their device while asleep. Finally, 11 participants were excluded as they did not report the demographic and behavioral data necessary for these analyses (Fig. 3C, D) and an additional 2207 sleep sessions were removed as physical activity data were missing for the previous day (Fig. 3E). At this stage, a cohort of 599 participants remained eligible for analysis.

### Normal bedtime

To determine how deviations from one’s normal bedtime may affect RHR, a definition for “normal bedtime” was first necessary. To provide a standard measure across participants, with minimal influence from outliers, the median of a participant’s bedtimes was chosen to represent their normal bedtime. However, utilizing a single median bedtime to represent multiple years of sleep habits is likely insufficient. Participant’s may have adjusted their sleep schedules over time in response to changes in class schedules across semesters or in absence of class schedules during the winter and summer breaks. To adjust for these potential variations, participant’s median bedtimes were computed within each of these respective time periods, specifically, for each semester and winter/summer break.

### Deviations from normal bedtime

With normal bedtimes established, bedtime deviations were then measured by calculating the difference (in min) between a participant’s normal bedtime and recorded bedtime for each respective night. To avoid the assumption of a linear relationship between the degree of bedtime deviation and RHR, bedtime deviations were discretized: binned into 11 categories to be examined separately. The first category provided our baseline, which was nights when the participant went to bed reasonably close to their median bedtime. We allotted a range of 30 min earlier to 30 min later than one’s normal bedtime to be defined as an on time bedtime. The remaining ten categories focused on deviations from this baseline, occurring earlier or later. The intervals for these deviation categories were [1, 30) min, [30, 60) min, [1, 2) h, [2, 3) h, and ≥3 h.

### Naps

Among the 311,267 total sleep sessions, 66,552 records (21%) occurred on the same day, likely indicating days in which a nap was taken. Although naps are commonly raised in literature, a formal definition regarding their duration or timing throughout the day remains to be agreed upon^36^. Further, as Fitbit devices do not allow for the annotation of sleep periods, an unsupervised approach was taken to more rigorously identify and remove such noise.

This was accomplished by utilizing a Variational Bayesian estimation of a Gaussian Mixture Model (BGMM) for clustering each sleep session by their duration and bedtime deviation. Gaussian mixture models (GMM) have repeatably demonstrated success in modeling data generated by an arbitrary number of distinct Gaussian processes. Which, as we have seen in prior processing steps, can reasonably be expected to hold true for the approximately normal distributions of each student’s sleep records. Further, as variational inference adds a level of regularization beyond traditional GMM approaches, the BGMM is able to provide a more robust estimate of the true underlying sleep patterns needed to remove the ambiguous notion of naps from the data. Moreover, to avoid making assumptions about the underlying nature of the sleep clusters, the BGMM was fit with full covariance structures, allowing each identified component to vary in both direction and shape.

**Fig. 4:**
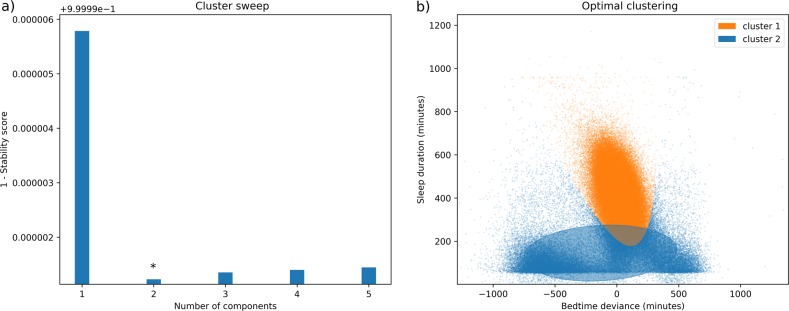
Clustering of sleep sessions by duration and deviation from normal bedtime. **a** The stability score as the number of components increases. **b** The distribution of sleep sessions for the optimal number of clusters, organized by sleep duration (*y*-axis) and deviation from normal bedtime (*x*-axis).

Finally, to evaluate the appropriate number of components (clusters) retrieved by the model, we defined a metric focused on rewarding the creation of distinct, nonoverlapping clusters in the data, a necessary addition, as Bayesian information criterion (BIC) is not applicable to BGMM. In an effort to identify stable, nonoverlapping clusters, we computed the average probability that each training point belonged to its respective cluster. To bound this, the average probability value is subtracted from 1, as the optimal situation would occur when every point is 100% likely to belong to its predicted cluster. Then, sweeping from 1 to 5 components, we selected the configuration that minimized this value, finding it to be 2 components (Fig. 4).

Among these two clusters, cluster 1 had a mean sleep duration of 7.03 h and mean bedtime difference of 1.04 h, while cluster 2 had a mean sleep duration of 2.26 h and mean bedtime difference of 6.7 h. Given the sleep sessions in cluster 2 had, on average, shorter durations and greater deviances from normal bedtimes, we removed sleep sessions within this cluster from our analysis as they more closely resembled characteristics of naps (Fig. 3f). In doing so, this also removed three participants, these participants likely only wore their device during naps, but removed the device before bed, failing to capture any full nights of sleep. Further, we removed the few remaining sleep sessions in which multiple sessions still occurred on the same day (Fig. 3g).

### Compliance

Finally, while NetHealth participants were followed for multiple years, not all participants were present for the full duration of the study. Reasons for this extended from participants entering the study late or having poor compliance to the study (not wearing their Fitbits) and abandonment of the study. To ensure participants with poor compliance would not bias the fixed effects of our models, we removed any participants with fewer than 30 sleep records, as having at least 30 units within each cluster is a commonly cited recommendation (Fig. 3H)^37^.

**Fig. 5:**
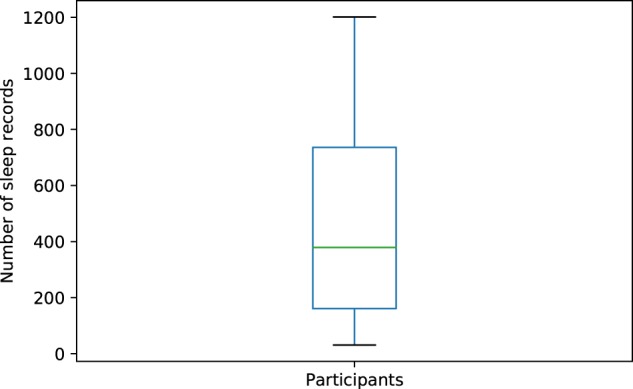
Boxplot of total sleep records contributed by participants. Boxplot statistics are as follows (Min = 31, Q1 = 161, Median = 379, Q3 = 736, Max = 1201).

Following our compliance threshold, our data set consisted of 557 participants and 255,736 sleep sessions upon which we conducted our analyses. A distribution of the total sleep records contributed by participants is provided in Fig. 5, with the interquartile range for number of records contributed as follows: (Q1 = 161 records, Q2 = 379 records, Q3 = 736 records).

### Resting heart rate

Our second research question focused on the amount of time for RHR to return to baseline, defined as the point in time when RHR following a bedtime deviation converged with RHR following no bedtime deviation. To ensure this return was fully captured, we examined, approximately, the 24-h period following one’s bedtime. Specifically, we monitored participant’s RHR beginning when they first went to bed until midnight of the following day. In doing so, it was necessary to examine RHR across periods when participants were asleep and awake. Given a variety of factors may influence RHR depending on when an individual is asleep or awake, we partitioned them into two separate analyses, taking two different approaches to best capture RHR for each state.

- Sleep: Beginning with sleep, we assumed sleep stages: light sleep, REM and deep sleep, to be one of the primary factors influencing changes in RHR over one’s sleep session^38,39^. To minimize the potential for differences in RHR to be the result of comparing participants at different sleep stages, all sleep sessions were aligned by bedtimes. Next, sleep sessions were partitioned by hour (first hour of sleep, second hour of sleep, etc.), allowing each hour of sleep to be examined separately. RHR was then measured by computing the median RHR for each hour of sleep within each nightly record. Sleep sessions were truncated at 7 h to avoid missing data resulting from variations in lengths of sleep sessions. Seven hours was chosen specifically, as this was the median sleep duration across participants. Finally, we note that in examining RHR across sleep, 14,877 sessions were missing RHR data, likely stemming from device failure rather than failure to wear the device, as bedtimes and sleep durations were still recorded (detected by the Fitbit accelerometer). As such, these sessions were excluded (Fig. 3I).
- Awake*:* As factors influencing RHR throughout one’s sleep are primarily relative to when one begins sleeping, the same does not hold for when one is awake. Specifically, factors influencing one’s RHR throughout the day are not necessarily relative to when one wakes up. Instead, individuals tend to keep with a daily social rhythm, where events correspond more to time of day, than when one wakes up^40,41^. For example, because one wakes up an hour later than usual, this does not necessarily mean they will also have lunch 1 h later than usual, as lunch time is determined more by time of day. Additional facets of these social rhythms can include adherence to a class schedule, leaving and returning from work, etc. which can affect RHR^40,41^. Therefore, when measuring RHR over the following day, we instead computed the median RHR for each hour of the day (9:00 am to 9:59 am, 10:00 am to 10:59 am, etc.) to better compare RHR across these social rhythms. Further, to capture RHR instead of HR, we only considered periods in which the participant had been inactive (no steps) for at least 30 min, in line with prior literature, with measurements ending when the participant began moving again^42–44^. Lastly, we note that in examining RHR across the following day, 51,368 days were excluded due to missing data as participants likely failed to wear their device throughout that day (Fig. 3J).

### Previous days’ physical activity

As detailed in the next section of the manuscript, we adjusted for several additional variables associated with RHR. Among these was the amount of physical activity the participant engaged in the day before, as physical activity has been shown to have an association with sleeping heart rate^45^. Fortunately, Fitbits also capture levels of physical activity based on heart rate readings. Fitbit partitions a user’s physical activity into three different heart rate zones based on the intensity of their activity: Fat Burn, Cardio, and Peak^46^. Therefore, we represent physical activity as the total amount of minutes a participant spent in any of these zones for each day. While a participant would predominantly spend their time in the Fat Burn range, as this can be accomplished through walking, this zone was included to adjust for the association of even light levels of physical activity, as any even light physical activity will increase heart rate.

Overall, our cohort eligibility requirements and preprocessing steps left us with two overlapping data pools: a total of 240,859 sleep sessions for assessing RHR over sleep and a total of 204,368 days for assessing RHR when awake over the following day.

### Modeling

To evaluate the association between bedtime deviations and RHR, we fit a series of linear mixed effects models, one for each hour of sleep and one for each hour of the following day up until midnight. This approach allowed us to assess both of our research questions simultaneously. For each hour following a deviation, we could examine whether differences in RHR were present (**RQ1**) and how long they persisted for (**RQ2**). Further, examining each hour independently allowed us to capture nonlinear trends in RHR over time. To supplement the models capturing these hourly level changes, we fit two additional mixed effects models for mean RHR: one over participants entire sleep session and the other over the following day; these models provided us a broad overview of the associations.

The decision to employ linear mixed effects models allowed us to account for the inter-instance correlation of sleep session data recorded multiple times for each individual. Further, they accounted for an additional source of variance. Specifically, the latent relationship between the time of day and an individual’s RHR moderated by their circadian rhythm.

A naturally occurring biological pattern, the circadian rhythm, dictates changes in the behavior and/or physiology of most species and has been shown to account for hourly variations in HR^38,41,47,48^. Unfortunately, rather than simply a global shift based on the absolute time of day, each individual can be expected to experience slightly differing effects at different times of day based on a broader consideration of their chronobiology^49^. As such, we extend the repeated measure mixed effects model to a multilevel model in which we nest observations for each absolute time of day under each individual. By accounting for variability in RHR by each hour of the day for a specific individual, we reduce the likelihood of finding a difference in two RHR measures because they were simply measured at different points of an individual’s circadian rhythm. Following this, we nest observations within individuals to account for the variability in RHRs between individuals. Ultimately, this approach provides a significantly more robust estimation of the unbiased effects of each covariate on the RHR based on deviations in bedtimes.

### Covariates

In addition to circadian rhythm, we also adjusted for several confounding variables, including features of one’s sleep session and external influences on RHR. External influences, such as sex and caffeine and alcohol consumption, were collected via the biannual surveys administered to participants. In the case of participants missing a survey, fill-forward/backward imputation was utilized, allowing these imputed values to most closely reflect participants true scores at the time and satisfy model requirements. We detail each of these variables below.

- Sleep duration—as prior work has associated insufficient sleep with increases in RHR, sleep duration was included in the model^50^.
- Naps—as some individuals may regularly account for sleep debt with naps in between nightly sessions, we include a binary flag for whether an individual had taken a nap over the previous day to adjust for this alternative sleep schedule.
- Sex—prior works have found differences in RHR between sexes with women having, on average, a higher RHR of 3–7 bpm^32^.
- Caffeine and alcohol consumption—we further adjusted for participants frequency of caffeine and alcohol consumption as both compounds have been found to be associated with increases in heart rate^32,33^.
- Prior day physical activity–as prior studies have found an association between physical activity and sleeping heart rate, the amount of physical activity engaged in the previous day was included in the model^45^.

The last covariate considered for the model was day of the week, as weekend nights may be more likely to bring about behaviors associated with increased sleeping heart rate, such as alcohol consumption. However, bedtime deviations and weekends are likely to be highly correlated given the natural tendency to stay up later on weekends, resulting in stronger bedtime deviations on the weekends. We find this assumption holds true among this cohort, as performing a chi-square test of independence on the frequencies of bedtime deviations stratified by weekday and weekend shows the difference between the distributions to be statistically significant (*P* < 0.001), with individuals going to bed later more frequently on the weekends.

To ensure increases in RHR were not solely a product of weekend behavior, we reconducted our analysis, this time using only weeknights (Sunday–Thursday). From our results, we observed similar changes in RHR using only weeknights suggesting the increases in RHR following a bedtime deviation are independent of weekend behaviors. Details for this analysis are provided in the supplementary materials: Supplementary Methods 2.

### Reporting summary

Further information on research design is available in the Nature Research Reporting Summary linked to this article.

## Supplementary information

Supplementary Information

Reporting Summary

## Data Availability

The data that support the findings of this study are available from the corresponding author upon reasonable request.

## Code availability

The code that support the findings of this study are available from the corresponding author upon reasonable request.
