# Supplementary material for: Deviations from normal bedtimes are associated with short-term increases in resting heart rate
Source: NPJ Digit Med. 2020 Mar 23;3:39. doi: 10.1038/s41746-020-0250-6 (PMC7090013; doi:10.1038/s41746-020-0250-6)
Supplement: Supplementary file 1 — Supplementary Information [file 41746_2020_250_MOESM1_ESM.pdf]

Supplementary Methods 1 – Compounding effects of consecutive bedtime deviations

Given the cyclical nature of sleep-wake states, we were interested in whether increases in RHR were associated with a single bedtime deviation or only the product of *consecutive* nights of bedtime deviations. To ensure increases in RHR were associated with a single night's bedtime deviation, we reconducted our analysis only considering nights (and the days that followed) that were preceded by an *on time* bedtime. This would allow the individual a period of recovery, having gone to bed on time the night before.

Supplementary tables 1 and 2 present the results from this analysis. We find that bedtime deviations, even after a night with a regular bedtime, were still associated with increases in RHR across the sleep session and into the following day. These observations suggest that increases in RHR are associated with an isolated deviation from one's normal bedtime.

Supplementary Table 1. Within-person differences in average resting heart rate during sleep across bedtime deviations only for weeknights (only nights followed by on time bedtime).

	**Earlier bedtimes[1,3]**	**Later bedtimes[2,3]**
**Bedtime deviation**	Coeff (95% CI)	Coeff (95% CI)
on time	*reference*	*reference*
[1, 30) minutes	0.04 (-0.08, 0.16)	0.45 (0.32, 0.58)
[30, 60) minutes	0.22 (0.06, 0.38)	0.94 (0.77, 1.11)
[1, 2) hours	0.55 (0.35, 0.75)	2.11 (1.91, 2.30)
[2, 3) hours	1.12 (0.72, 1.51)	3.39 (3.09, 3.69)
≥ 3 hours	2.92 (2.26, 3.58)	3.89 (3.35, 4.43)

[1]N = 554 participants, 46,055 nights

[2]N = 554 participants, 51,877 nights

[3]Coefficients are representative of differences in resting heart rate bpm adjusting for sleep duration, naps, sex, prior day's physical activity and frequency of caffeine and alcohol consumption.

Supplementary Table 2. Within-person differences in average resting heart rate during the next day across bedtime deviations only for weeknights (only nights followed by on time bedtime).

	**Earlier bedtimes[1,3]**	**Later bedtimes[2,3]**
**Bedtime deviation**	Coeff (95% CI)	Coeff (95% CI)
on time	*reference*	*reference*
[1, 30) minutes	0.03 (-0.13, 0.20)	0.19 (0.03, 0.36)
[30, 60) minutes	0.09 (-0.14, 0.32)	0.18 (-0.02, 0.39)
[1, 2) hours	0.38 (0.01, 0.66)	0.57 (0.35, 0.79)
[2, 3) hours	0.44 (-0.14, 1.03)	0.83 (0.47, 1.19)
≥ 3 hours	0.78 (-0.21, 1.78)	0.93 (0.20, 1.67)

[1]N = 545 participants, 40,277 days

[2]N = 545 participants, 45,044 days

[3]Coefficients are representative of differences in resting heart rate bpm adjusting for sleep duration, naps, sex, prior day's physical activity and frequency of caffeine and alcohol consumption.

Supplementary Methods 2 – Bedtime deviations and weekend behaviors

As one can reasonably assume, bedtime deviations are more likely to occur on weekends. While this association alone is unlikely to affect resting heart rate (RHR), confounding weekend behaviors such as alcohol consumption may also be associated with increases in RHR. To ensure increases in RHR were not solely a product of weekend behavior, we reconducted our analysis only considering weeknights (Sunday – Thursday) to remove any association with weekend behavior.

Supplementary Tables 3 and 4 present the results from this analysis. We find that bedtime deviations occurring on weeknights are also associated with increases in RHR through the sleep session and into the following day. These observations suggest, the association between bedtime deviations and RHR is independent of weekend behavior.

Supplementary Table 3. Within-person differences in average resting heart rate during sleep across bedtime deviations only for weeknights (Friday and Saturday nights withheld).

	**Earlier bedtimes[1,3]**	**Later bedtimes[2,3]**
**Bedtime deviation**	Coeff (95% CI)	Coeff (95% CI)
on time	*reference*	*reference*
[1, 30) minutes	0.09 (0.01, 0.17)	0.16 (0.08, 0.24)
[30, 60) minutes	0.26 (0.16, 0.36)	0.48 (0.37, 0.58)
[1, 2) hours	0.65 (0.55, 0.76)	1.04 (0.92, 1.16)
[2, 3) hours	1.20 (1.01, 1.38)	2.06 (1.88, 2.24)
≥ 3 hours	2.71 (2.44, 2.99)	2.92 (2.63, 3.22)

[1]N = 557 participants, 115,494 nights
[2]N = 557 participants, 116,545 nights
[3]Coefficients are representative of differences in resting heart rate bpm adjusting for sleep duration, naps, sex, prior day's physical activity and frequency of caffeine and alcohol consumption.

Supplementary Table 4. Within-person differences in average resting heart rate during the next day across bedtime deviations only for weeknights (Friday and Saturday nights withheld).

	**Earlier bedtimes[1,3]**	**Later bedtimes[2,3]**
**Bedtime deviation**	Coeff (95% CI)	Coeff (95% CI)
on time	*reference*	*reference*
[1, 30) minutes	-0.01 (-0.12, 0.09)	-0.001 (-0.11, 0.11)
[30, 60) minutes	0.02 (-0.10, 0.16)	0.04 (-0.08, 0.17)
[1, 2) hours	0.21 (0.06, 0.35)	0.13 (-0.003, 0.26)
[2, 3) hours	0.35 (0.08, 0.61)	0.42 (0.21, 0.63)
≥ 3 hours	0.31 (-0.09, 0.72)	0.92 (0.53, 1.32)

[1]N = 557 participants, 99,993 days
[2]N = 557 participants, 101,051 days
[3]Coefficients are representative of differences in resting heart rate bpm adjusting for sleep duration, naps, sex, prior day's physical activity and frequency of caffeine and alcohol consumption.

Supplementary Table 5. Results from a Linear Mixed Effects model detailing differences in sleeping resting heart rate across covariates

		Coeff (95% CI)
**Sex**		
	Male	*reference*
	Female	4.87 (3.77, 5.97)
**Caffeine**		
	Not at all	*reference*
	Less than 1-2 times a month	-0.08 (-0.03, 0.19)
	1-2 times a month	0.01 (-0.10, 0.12)
	1-2 times a week	0.22 (0.11, 0.33)
	Three times a week or more	0.22 (0.10, 0.34)
**Alcohol**		
	Not at all	*reference*
	Less than 1-2 times a month	-0.10 (-0.21, -0.004)
	1-2 times a month	0.11 (0.005, 0.22)
	1-2 times a week	0.20 (0.09, 0.31)
	Three times a week or more	0.49 (0.32, 0.66)
**Physical activity (hours)**		1.30 (1.28, 1.31)
**Sleep duration (hours)**		-0.03 (-0.05, -0.01)

N = 557 participants, 240,859 records

Supplementary Table 6. Differences in resting heart rate compared to going to bed on time across seven hours of sleep

Bedtime deviation
Coeff (95% CI)

Later bedtimes, N = 557 participants, 166,292 nights

**Hour of sleep**	on time	[1, 30) minutes later	[30, 60) minutes later	[1, 2) hours later	[2, 3) hours later	≥ 3 hours later
first hour	*reference*	0.06 (-0.03, 0.16)	0.42 (0.30, 0.54)	0.96 (0.83, 1.09)	1.65 (1.45, 1.84)	2.31 (1.99, 2.63)
second hour	*reference*	0.14 (0.04, 0.23)	0.48 (0.37, 0.60)	0.99 (0.86, 1.11)	1.87 (1.68, 2.05)	2.47 (2.16, 2.77)
third hour	*reference*	0.14 (0.06, 0.23)	0.52 (0.41, 0.63)	1.08 (0.96, 1.20)	1.99 (1.82, 2.17)	2.81 (2.56, 3.11)
fourth hour	*reference*	0.15 (0.07, 0.23)	0.53 (0.43, 0.63)	1.15 (1.04, 1.27)	2.15 (1.98, 2.32)	2.99 (2.71, 3.27)
fifth hour	*reference*	0.27 (0.19, 0.35)	0.72 (0.63, 0.82)	1.35 (1.24, 1.46)	2.47 (2.30, 2.64)	3.27 (2.99, 3.54)
sixth hour	*reference*	0.32 (0.24, 0.40)	0.80 (0.70, 0.90)	1.60 (1.49, 1.71)	2.68 (2.50, 2.86)	3.51 (3.22, 3.80)
seventh hour	*reference*	0.44 (0.36, 0.52)	1.00 (0.89, 1.11)	1.78 (1.65, 1.90)	2.64 (2.44, 2.84)	3.16 (2.82, 3.50)

Earlier bedtimes, N = 557 participants, 153,385 nights

**Hour of sleep**	on time	[1, 30) minutes earlier	[30, 60) minutes earlier	[1, 2) hours earlier	[2, 3) hours earlier	≥ 3 hours earlier
first hour	*reference*	0.27 (0.18, 0.36)	0.73 (0.61, 0.84)	1.44 (1.31, 1.56)	2.63 (2.41, 2.84)	4.61 (4.29. 4.93)
second hour	*reference*	0.11 (0.03, 0.20)	0.39 (0.29, 0.50)	0.97 (0.85, 1.09)	1.72 (1.52, 1.93)	3.34 (3.04, 3.64)
third hour	*reference*	0.06 (-0.01, 0.14)	0.31 (0.21, 0.41)	0.74 (0.63, 0.85)	1.37 (1.18, 1.56)	2.90 (2.62, 3.18)
fourth hour	*reference*	0.03 (-0.04, 0.11)	0.25 (0.15, 0.34)	0.61 (0.50, 0.71)	1.06 (0.88, 1.24)	2.42 (2.15, 2.68)
fifth hour	*reference*	-0.02 (-0.09, 0.05)	0.14 (0.05, 0.23)	0.39 (0.29, 0.50)	0.85 (0.67, 1.02)	2.01 (1.84, 2.35)
sixth hour	*reference*	-0.11 (-0.18, -0.04)	-0.05 (-0.14, 0.03)	0.17 (0.06, 0.27)	0.56 (0.39, 0.72)	1.33 (1.08, 1.58)
seventh hour	*reference*	-0.17 (-0.24, -0.10)	-0.13 (-0.22, -0.04)	-0.01 (-0.11, 0.09)	0.14 (-0.02, 0.31)	0.80 (0.55, 1.05)

Coefficients are representative of differences in resting heart rate adjusting for sleep duration, naps, sex, prior day physical activity and frequency of caffeine and alcohol consumption.

Supplementary Table 7. Differences in resting heart rate compared to going to bed on time across the following day

	Bedtime deviation<br>Coeff (95% CI)					
**Later bedtimes**, N = 557 participants, 131,079 days						
**Time of day**	on time	[1, 30) minutes later	[30, 60) minutes later	[1, 2) hours later	[2, 3) hours later	≥ 3 hours later
9am	*reference*	-0.36 (-0.58, -0.14)	-0.51 (-0.78, -0.24)	-0.28 (-0.59, 0.02)	-0.02 (-0.55, 0.50)	0.32 (-1.02, 1.67)
10am	*reference*	-0.004 (-0.19, 0.19)	0.11 (-0.12, 0.34)	0.45 (0.20, 0.71)	1.25 (0.82, 1.68)	1.02 (0.04, 2.01)
11am	*reference*	0.41 (0.23, 0.60)	0.75 (0.53, 0.97)	1.14 (0.91, 1.37)	1.74 (1.36, 2.12)	3.11 (2.31, 3.91)
12pm	*reference*	0.20 (0.01, 0.39)	0.41 (0.18, 0.63)	0.94 (0.71, 1.17)	1.47 (1.10, 1.84)	1.76 (1.02, 2.50)
1pm	*reference*	0.08 (-0.10, 0.26)	0.11 (-0.10, 0.33)	0.07 (-0.15, 0.29)	0.85 (0.50, 1.20)	1.21 (0.51, 1.90)
2pm	*reference*	0.14 (-0.03, 0.32)	-0.01 (-0.21, 0.19)	0.13 (-0.07, 0.34)	0.91 (0.58, 1.24)	0.90 (0.27, 1.52)
3pm	*reference*	0.04 (-0.13, 0.22)	0.06 (-0.15, 0.27)	0.20 (-0.01, 0.42)	0.63 (0.29, 0.97)	1.33 (0.71, 1.95)
4pm	*reference*	0.04 (-0.15, 0.23)	0.21 (-0.01, 0.43)	0.11 (-0.11, 0.34)	0.48 (0.12, 0.83)	0.84 (0.18, 1.49)
5pm	*reference*	-0.20 (-0.42, 0.01)	-0.14 (-0.39, 0.11)	0.01 (-0.25, 0.26)	0.49 (0.09, 0.89)	0.74 (-0.01, 1.49)
6pm	*reference*	-0.28 (-0.52, -0.05)	-0.11 (-0.39, 0.15)	-0.22 (-0.49, 0.05)	-0.12 (-0.54, 0.29)	0.26 (-0.51, 1.04(
7pm	*reference*	-0.24 (-0.44, -0.03)	-0.08 (-0.32, 0.16)	-0.27 (-0.52, -0.03)	-0.04 (-0.41, 0.33)	-0.46 (-1.15, 0.22)
8pm	*reference*	-0.14 (-0.34, 0.05)	-0.11 (-0.34, 0.11)	-0.27 (-0.50, -0.04)	-0.48 (-0.83, -0.12)	-0.41 (-1.05, 0.23)
9pm	*reference*	-0.07 (-0.27, 0.12)	-0.01 (-0.23, 0.21)	-0.01 (-0.23, 0.21)	-0.16 (-0.51, 0.18)	-0.27 (0.91, 0.38)
10pm	*reference*	0.01 (-0.18, 0.21)	0.03 (-0.19, 0.26)	0.01 (-0.22, 0.23)	-0.20 (-0.56, 0.16)	-0.27 (-0.94, 0.38)
11pm	*reference*	0.03 (-0.17, 0.24)	0.01 (-0.23, 0.24)	-0.05 (-0.29, 0.19)	-0.001 (-0.38, 0.38)	-0.18 (-0.88, 0.51)
12am	*reference*	0.14 (-0.10, 0.38)	0.08 (-0.19, 0.35)	0.37 (0.10, 0.65)	0.37 (-0.05, 0.79)	-0.14 (-0.91, 0.62)
**Earlier bedtimes**, N = 557 participants, 141,511 days						
**Time of day**	on time	[1, 30) minutes earlier	[30, 60) minutes earlier	[1, 2) hours earlier	[2, 3) hours earlier	≥ 3 hours earlier
9am	*reference*	0.27 (0.05, 049)	0.47 (0.20, 0.75)	0.21 (-0.10, 0.52)	0.25 (-0.32, 0.83)	1.21 (0.31, 2.11
10am	*reference*	-0.08 (-0.27, 0.11)	-0.003 (-0.24, 0.24)	-0.05 (-0.33, 0.21)	0.52 (0.02, 1.03)	0.37 (-0.42, 1.16)
11am	*reference*	-0.01 (-0.19, 0.17)	0.23 (0.005, 0.46)	0.02 (-0.23, 0.28)	0.40 (-0.06, 0.87)	0.26 (-0.48, 1.00)
12pm	*reference*	-0.02 (-0.21, 0.16)	-0.04 (-0.27, 0.19)	-0.01 (-0.28, 0.24)	-0.16 (-0.64, 0.32)	0.53 (-0.20, 1.26)
1pm	*reference*	-0.01 (-0.20, 0.17)	0.13 (-0.10, 0.36)	0.22 (-0.04, 0.48)	0.11 (-0.37, 0.59)	1.16 (0.44, 1.89)
2pm	*reference*	0.15 (-0.02, 0.33)	-0.03 (-0.25, 0.18)	0.43 (0.19, 0.67)	0.88 (0.43, 1.33)	1.59 (0.91, 2.27)
3pm	*reference*	0.08 (-0.10, 0.26)	0.20 (-0.02, 0.43)	0.61 (0.35, 0.86)	0.35 (-0.11, 0.82)	0.99 (0.27, 1.70)
4pm	*reference*	0.03 (-0.16, 0.23)	0.31 (0.07, 0.56)	0.55 (0.28, 0.81)	0.74 (0.26, 1.23)	0.54 (-0.20, 1.28)
5pm	*reference*	0.03 (-0.19, 0.26)	0.05 (-0.22, 0.32)	0.42 (0.12, 0.72)	0.63 (0.10, 1.16)	0.34 (-0.48, 1.16)
6pm	*reference*	-0.23 (-0.47, 0.003)	-0.33 (-0.63, -0.05)	-0.12 (-0.41, 0.18)	-0.01 (-0.56, 0.53)	0.54 (-0.29, 1.38)
7pm	*reference*	-0.13 (-0.35, 0.07)	-0.22 (-0.48, 0.03)	-0.43 (-0.71, -0.15)	-0.57 (-1.06, -0.08)	0.03 (-0.72, 0.79)
8pm	*reference*	-0.26 (-0.46, -0.06)	-0.24 (-0.48, -0.06)	-0.32 (-0.58, -0.06)	-0.32 (-0.78, 0.14)	-0.14 (-0.86, 0.56)
9pm	*reference*	-0.23 (-0.43, -0.04)	-0.33 (-0.57, -0.09)	-0.52 (-0.78, -0.26)	-0.41 (-0.87, 0.04)	-0.45 (-1.17, 0.27)
10pm	*reference*	-0.32 (-0.53, -0.12)	-0.54 (-0.79, -0.29)	-0.74 (-1.01, -0.47)	-0.99 (-1.47, -0.50)	-0.99 (-1.73, -0.24)
11pm	*reference*	-0.34 (-0.56, -0.11)	-0.41 (-0.68, -0.15)	-0.64 (-0.93, -0.35)	-0.27 (-0.81, 0.26)	0.05 (-0.73, 0.84)
12am	*reference*	-0.21 (-0.47, 0.05)	0.30 (-0.01, 0.62)	0.06 (-0.28, 0.41)	-0.11 (-0.74, 0.51)	0.93 (0.02, 1.84)

Coefficients are representative of differences in resting heart rate adjusting for sleep duration, naps, sex, prior day physical activity and frequency of caffeine and alcohol consumption.
